# “You can definitely tell she's truly committed”: student-athletes’ discursive construction of athletic commitment during transitions in secondary school

**DOI:** 10.3389/fspor.2025.1658234

**Published:** 2025-10-07

**Authors:** Marie Loka Øydna, Milla Saarinen, Jens Christian Nielsen, Christian Thue Bjørndal

**Affiliations:** ^1^ The Child and Youth Sport Research Center, Norwegian School of Sport Sciences, Oslo, Norway; ^2^ The Danish Institute for Cultural Policy Analysis, Roskilde, Denmark; ^3^ Department of Sport and Social Sciences, Norwegian School of Sport Sciences, Oslo, Norway

**Keywords:** Foucault, talent development, dual career, athletic identity, youth athletes

## Abstract

In this paper, we present how two female and one male student-athlete discursively construct and make sense of the “committed athlete”, highlighting key shifts across transitions between school levels and high-performance sport environments. Applying a Foucauldian perspective on power/ knowledge and vignettes from follow-up interviews, this paper highlights the relational nature of commitment, showing how it is shaped by broader discourses and institutional structures. Our analysis shows that a discourse of athletic performance investment dominates these transitions, yet the student-athletes also negotiate less instrumentalised constructions of the committed athlete as someone with internal love for the activity itself. However, as they move into upper secondary school programmes and more professionalised club environments, they are faced with fewer possibilities to enact alternative meanings of commitment. We argue that student-athletes positioned as talented are better able to benefit from holistic development initiatives within the talent development system, while those outside these hierarchies face limited possibilities to negotiate alternative forms of commitment. This analysis invites a critical rethinking of how dual career pathways might expand the discursive possibilities for what it means to be a committed athlete.

## 1 Prologue

**Theodor** slid another weight onto the bar, the metal clinking like a challenge. “Come on, we’ve got this”, he said to Sivert, his voice steady, like he'd already decided for both of them. “Mate, imagine if we hit a new PB. Just think about it”. There was no stopping Theodor. There never was.

**Elise** sat on the floor, her back pressed against the wall, a resistance band looped around her ankle. She moved slowly, controlled, mechanical. Another week, another session, and still no real progress. A year of this. A whole year of following through injury routines while everyone else kept developing.

**June** stood at the edge of the air track, hands on her hips, watching the boys show off. “Watch this!” she called, loud enough to make the football girls glance up. She sprinted, jumped, and went for the front flip—but her heels hit first, and she tumbled onto her back. The thud hung in the air before her laughter broke it. “Almost nailed it!” she shouted, brushing off her too-big Nike track pants, with the national team's logo barely visible in the folds.

This paper is part of the first author's doctoral research, in which fieldwork was conducted at one particular lower secondary sports school, and she constructed the data material through various methods, such as document analysis, participant observations, informal conversations, and semi-structured interviews. These creative narratives were constructed from her observational notes during a school training session in a gymnastics hall in the fall of 2022, recollecting her first interpretations of Theodor, Elise, and June (pseudonyms). At that time, they were between 14 and 15 years old, and all shared the aspiration of becoming elite athletes in their respective sports.

## 2 Introduction

Transitions in high-performance junior or senior sport are increasingly recognised as critical periods that shape young athletes' developmental, educational, and psychosocial trajectories (1). These transitions, whether between school levels, sport environments, or stages of maturation, are not merely logistical shifts but are laden with discursively constructed expectations about what it means to be simultaneously a committed athlete, autonomous citizen, and gendered individual (2, 3). Such periods are often marked by tensions, overlaps, and reconfigurations between multiple identities, all shaped by power relations and institutional structures within the athlete's broader context (4, 5). Although existing research has significantly advanced our understanding of dual career pathways and transitional processes, there is a need for more context-sensitive studies that explore how young athletes work to sustain meaningful identities across changing environments. This is particularly relevant as athletes begin to engage more seriously with performance issues, while still holding on to fun and enjoyment as core motivational drivers (6).

Throughout, a growing body of theoretical perspectives, research, and practice on supporting transitioning athletes eventually grew into an European dual career discourse, highlighting the need to facilitate high performance sport participation in a socially responsible manner by promoting the simultaneous pursuit of studies or work (7). A key focus in Norwegian talent development has therefore been to secure professionalised and structured pathways that support both athletic development and education through specialised sport school programmes (8, 9). Recent developments have extended similar opportunities into lower secondary education, marking a significant shift in how early young people enter environments where athletic and academic pathways are structured.

These structures are often taken for granted and not examined for how they position the student-athletes within competing discursive realities and power relations (10, 11). Studies have, for example, problematised dual career environments for normalising additional expectations of athletes, requiring them to achieve even more despite already facing significant pressures on time and energy (12, 13). Researchers have also suggested that dual career pathways are often structured around athletic performance, creating tension for coaches and policymakers to support student-athletes in adopting a more holistic approach to their athletic careers (14–16). For example, emotional distress, injuries, overuse, and burnout are common risks for young student-athletes, while an overemphasis on sport also tends to skew the dual focus (17, 18).

Within these environments, sociocultural discourses of high-performance sport that celebrate athletic commitment are often privileged [e.g., (19–21)]. Commitment is typically positioned as a moral virtue and performance imperative, often demonstrated through discipline, delayed gratification, and adherence to expert-driven performance regimes (22, 23). These regimes are underpinned by bioscientific knowledge systems that normalise the idea of the athletic body as a site of constant optimisation (24). Consequently, athletes are interpellated into self-governing practices that prioritise productivity, bodily control, and resilience, leaving little space for alternative ways of being or resisting dominant norms (25). Moreover, by positioning athletes as morally obligated to immerse themselves in the quest for excellence, reinforcing morality-based fears surrounding the “it's really up to you” doctrine permeating scientific discourses on athletes' health and development (26), individualised solutions are favoured in holistic development campaigns over structural solutions (2, 11, 27).

Several researchers have used social theory to illuminate how older athletes make sense of and manage their identities over time and within or outside the justification of a sporting system [e.g., (28–30)]. Departing from these ideas, we employ poststructural theory and Foucault's power/knowledge framework to explore the discursive resources available to younger athletes during transitions between dual career development environments. By giving recognition to the “work” young athletes do to position as committed athletes within shifting power relations of talent development practice, we can shed light on the ways in which their local context privileges or complicates such transition processes. Specifically, we ask two questions: (a) How do student-athletes' discursive constructions of commitment change over time and across educational transitions? and (b) What do these shifts reveal about the discursive possibilities for practicing commitment in high-performance junior sport contexts?

To address the first question, we trace the specific discourses through which the student-athletes construct and make sense of the “committed athlete”, highlighting key shifts across time and context. The second question is taken up in the discussion, where we juxtapose the vignettes of Theodor, Elise, and June to explore how the imperative to invest in sport is both normalised and negotiated and how, in different ways, they navigate, resist, or reproduce the disciplinary demands of high-performance environments.

In doing so, we contribute to the growing literature that critically examines the ethical and political dimensions of athlete transitions (3, 30–32). Our aim is not only to trace how the committed athlete is constructed but also to consider what is *made possible or foreclosed* by these constructions, especially for young athletes navigating systems that both enable and constrain development. The study also foregrounds the implications for coaches and educators working within increasingly professionalised youth sport environments, highlighting how discursive flexibility—or a lack thereof—shapes young people's experiences of sport and education.

## 3 Theoretical perspectives: power relations and discourse

Our work draws upon the writings of Foucault. The work of Shogan (33), Markula and Pringle (34), and Avner et al. (35) have extended these ideas into sports coaching and are especially relevant for examining how possibilities for athlete development are constructed. Foucault's primary focus was not on defining the essence or origin of power but on examining how power operates dynamically and relationally. Foucault (36) asserts that the effects of power materialise through actions: “[It] does not act directly (…) it acts upon actions: action upon an action” (p. 789). His conception of power moves beyond a dualistic, top-down framework, positioning it as inseparable from the scientific truth-production of knowledge.

Foucault (37) uses the notion of discourse to describe dominant ways of knowing a particular social field (e.g., talent development), as well as understanding the dominant practices that establish what is constructed as reality:

The ensemble of more or less regulated, more or less deliberate, more or less finalized ways of doing things, through which can be seen both what was constituted as real for those who sought to think it and manage it and the way in which the latter constituted themselves as subjects capable of knowing, analyzing, and ultimately altering reality [(37). p. 463]

For example, practices that reproduce discourses of elite sport, such as writing training journals and tracking recovery, acquire their significance from being interpreted within the perspective of improved performance (38). These practices are implemented to generate knowledge about the athletic body, which then places an athlete in dependence with culturally or scientifically accepted standards that imply particular expectations as to how one should act upon this knowledge. These discursive practices thus provide the limits and possibilities for athletes and young people to produce meaning about sport participation and their sense of self (39).

No discourses are in themselves “good” or “bad”, but they become problematic when the beliefs and truth-presumptions are naturalised and no longer questioned for their productivity (35). Within a socio-historical context where multiple discourses circulate (e.g., related to age, body size, health, or gender), tensions can emerge as certain “truth-games” meet and clash, complicating the construction of a coherent sense of self and the negotiation of acceptable ways of being an athlete (5, 40). For instance, female student-athletes are expected to be autonomous, self-confident, and tough, attributes that are in contrast to how women in general are expected to behave (13). Hence, when looking at changes in how adolescents discursively construct meanings of commitment, it is important to trace how different discursive elements are organised and interconnected to render what effects these constructions have for adolescents' understanding of their moral obligations and self-governing practices (41, 42). This is central to revealing the power relations involved in the production of certain subject positions and understanding of the self (that is, positions in discourses that represent ways of being an individual) as privileged, more accessible, or constructed as “natural”, while others are deemed “unnatural” or marginalised. In a talent development context, taking up or resisting privileged positions influences who is prioritised and granted access to resources or authority (43, 44).

## 4 Methodology

In this paper, we approach knowledge and meaning as constructed through interactions between participants and researchers, shaped by the social, historical, and political context of which the interaction is practiced (45). From this position, interviews are seen as dynamic discursive spaces where participants actively construct and articulate their understandings based on the subject positions made available to them (46). As such, we consider researchers' reflexivity, vignettes close to the student-athletes’ own descriptions, and personal engagement with a dual career trajectory as important tools for deepening our understandings of the power relations shaping the lives of student-athletes (47).

### 4.1 The Norwegian context

The organising of talent development in Norway diverges distinctively from national elite sport systems or academy-based models prevalent in non-Scandinavian countries (48). In a country where football (soccer) and handball dominate as the most popular team sports among adolescents, significant resources are directed towards talent management systems designed to optimise player development and improve national performance outcomes (49). Within this structure, sports school programmes play a central role in better accommodating the dual career aspirations of student-athletes, operating autonomously while coordinating with club teams and sports associations, including age-specific regional and national teams (8). These schools are organised around a dual career policy request, that is, to arrange for athletes to engage in elite sport practices that make them capable of winning medals in the long run, and at the same time qualify for university and academic education (50). While Norway has a long-standing tradition of offering dual career programmes, including both elite sport and sports-friendly tracks (51), at the upper secondary level,^1^ recent developments have extended similar opportunities into lower secondary education.

The Norwegian Directorate of Education oversees 23 lower secondary sports schools, educating over 4,000 students aged 12 to 16 years, corresponding to grades 8 through 10. These schools, although operating as private institutions, receive 85% of their funding from the government and are required to follow the standard lower secondary school curriculum. Athlete development within these schools is integrated into education-focused training sessions, guided by the long-term athlete development framework that heavily shapes the policy documents governing Norwegian talent development. Their educational aim is to develop student-athletes' general athletic abilities, sport-specific skills, and the routines and mindsets that are considered essential to ensuring success in the competitive realm of elite sports (11).

### 4.2 The research process

The first author conducted three semi-structured interviews with Theodor and Elise: the first interview was conducted at their school in the autumn when they were 15 years old, followed by a second interview in the spring of their 10th-grade year. The third interview took place at a location of the participants' own choosing in late autumn the following year, after they had transitioned to upper secondary school and turned 16. June participated only in the two autumn interviews due to her busy schedule. No coaches or guardians supervised the interviews. The focus in each interview was to enquire how the participants came to speak of themselves as committed athletes. Each interview was designed to locate the participants within the discursive practices of talent development, which involved the first author calling upon them to differentiate, compare, and scrutinise elements of their lives in relation to established norms for long-term performance development, personal restitution (e.g., adequate nutrition and sleep), and healthcare (e.g., injury prevention and training load management).

Furthermore, in each interview, the participants were asked about their relationship with their school, what they did or did not like, how they perceived the social relations in their class, how they had benefitted from the educational programme, and how they had coordinated school activity with their club activity. Since the last interview was conducted after they had transitioned to upper secondary education, we focused more on the differences between the educational programmes and the changes they had experienced when we designed the third interview guide than in the two former interviews. Each interview lasted 45–75 min, was audio-recorded using a digital recording device, transcribed verbatim, and immediately anonymised for storage and analysis.

### 4.3 Ethical considerations

The study received ethical approval from both the Norwegian Agency for Shared Services in Education and Research (reference number 618455) and the Ethics Review Board of the Norwegian School of Sport Sciences. Prior to conducting the interviews, the first author obtained informed consent electronically from both the student-athletes and at least one of their parents. They received their interview transcripts via e-mail and were informed that any information they did not want disclosed could be excluded from the transcripts. They were also informed that they could withdraw from the study at any point without consequences. All the participants were assured they would be given pseudonyms, and that references to coaches, teachers, or peers would be anonymised. In crafting the vignettes, we diligently balanced specific details with general characteristics to value the participants' privacy, ensuring as much confidentiality as possible.

While the first author did not attend a lower secondary sports school, she transitioned into a dual career development environment at the age of 16, navigating the demands of balancing athletic and academic commitments during several transitional stages. Her underlying assumptions about commitment and her position as a student-athlete balancing elite handball with her PhD studies were meaningful points of reflection throughout the research process. For example, during the interviews, she actively challenged some taken-for-granted assumptions about athlete development to distinguish herself from coaches and other experts whose job is to sharpen the student-athlete's awareness about “best practice”. As an insider to the elite sport culture, the first author shared a similar schedule and understanding of social practices with the participants, whereas her subjectivity was also constructed through her negotiation between oppression and pleasure as two intrinsically linked entities. However, her insider status also created distinct power relations in the interviews when she made them accountable for why they saw themselves as committed athletes (52). Nevertheless, our focus is not on uncovering an objective “Truth” (e.g., whether they *are* committed) but rather on tracing the knowledge system, or what is “*within the true*” (e.g., what is constructed as legitimate practices of commitment) (53). These dynamics, therefore, can offer insights into the broader taken-for-granted assumptions inherent in Norwegian talent development.

The second, third, and fourth authors are experienced researchers whose expertise spans across youth studies, educational and sport psychology, and youth athlete development, and who were also significant in shaping the results. The fourth author's previous experiences as a teacher, and coach, and manager within the Norwegian sport school sector, the talent development pathway, and elite handball further substantiated our contextual understanding and interpretations. Through discussions, the second, third, and fourth authors acted as critical friends, challenging the first author's assumptions and interpretations. This collaborative process was crucial for assessing whether the vignettes provided meaningful insights into the social lives of student-athletes and ensuring that the analysis aligned with the study's underlying philosophical framework.

### 4.4 Analysis

We followed the recommendations of Bacchi and Bonham (54) on how to carry out a Foucault-influenced poststructural interview analysis. First, the first author noted references to commitment, paying attention to both direct and indirect statements about becoming athletes, such as discussions of future careers, training, and how sport intersected with school and social life. These insights were integrated into the construction of vignettes. Second, to trace the discourses available to student-athletes, we examined when and where commitment was discussed. For instance, Elise associated commitment with training sessions and personal motivation, while Theodor related it primarily to self-discipline outside of training. We then connected these statements to elements that rendered it difficult for student-athletes to construct commitment differently. For example, the objectifying gaze of the national team classified June as a talent, positioning her within tailored training schedules where commitment became intertwined with performance enhancement. This led us to trace their statements back to discourses already located in the scientific literature and to dominant knowledge systems (e.g., sport physiology and psychology) and related norms (e.g., about training loads). Finally, we linked these knowledge systems and practices to the power relations of talent development, identifying the discursive resources available for constructing and enacting commitment during transition processes.

### 4.5 Presentation strategy: vignettes

We decided to present a layered account of the participants' complex and nonlinear experiences of transitions between dual career development environments through a set of vignettes (55). The vignettes were constructed to provide the reader with a sense of the participants' own telling—insofar as the authors can ever create accounts that feel truthful and close. From the myriads of statements made during the interviews, it is us who have chosen the core of Theodor's, Elise's, and June's stories. From a practitioner's perspective working with athlete development, June's transition can be seen as a “success” story about fulfilling the dual career ideal of balancing elite sport and education, and the subsequent happy and rewarding engagement with the talent development system. In contrast, Elise's transition can, for example, be interpreted as a story about her struggling to advance her sporting performance while also trying to fit in with the prevailing norms of youth culture. Similarly, Theodor can be seen as someone who diligently follows the expected pathways to achieve future success but continues to struggle for recognition within a system that prioritises current performance over potential.

Indeed, from our research position, which purports the coexistence of multiple social realities, we perceive such an interpretation to be a “valid” one. At the same time, however, if we pose questions from a critical sociocultural perspective, such as “What processes have constrained their talent development?” or “What and in whose interest does this type of talent development serve?”, the stories immediately become more complex. Hence, the vignettes were purposefully made to integrate the myriads of experiences involved in the student-athletes' lives, within and beyond sport, to highlight the changes in conditions shaping their transitions. Each vignette was subsequently enriched with a more focused discursive analysis of how each athlete constructed the notion of commitment, allowing us to illustrate the parallel shifts in these constructions over time. Our Foucauldian reading of the key implications of their stories, and the shifting power relations that shaped them, is reserved for the discussion.

## 5 Transition vignettes

The following vignettes shed light on lives lived across time to avoid episodic focuses on single transitions (28). To assist in building our argument that identity constructions are fluid and relational, and to demonstrate that a transition process inevitably produces changes in other spheres of life, we present the student-athletes' experiences of identity management set against the context of their lives. We contend that this perspective offers an alternative to outcome-oriented research, which often obscures the “production process” of transition experiences in favour of static, descriptive accounts.

### 5.1 Theodor: manoeuvring investment and the promise of future returns

A focal point in Theodor's story was his non-selection for the elite sports upper secondary school programme he had aspired to. His educational transition proved challenging, shaped by heightened expectations and reduced support from new teachers. He also questioned the competence of his new school coaches, expressing doubt about whether they could provide the level of guidance he needed to support his athletic development. Despite maintaining strong performance at the club level and retaining his position as a first-team player, he was not re-selected to the Norwegian Handball Federation's Talent Pathway. The transition was therefore experienced as difficult and destabilising. In reflecting on the significance of his athletic ambitions and his friendships within handball, his non-sporting identities played a less prominent role in how he navigated and managed this transitional phase.

December 2023

I have three plans in life: handball, handball, and handball. My mom thinks I should have a backup plan, but at the same time, she gets where I'm coming from. She once told me, “You know, I struggled with school and motivation too”. That's why I like this school, because they help me improve. The teachers understand us, and they don’t give us any homework here, which makes everything more chill. School's tough for me, and to be honest, I don't find it that interesting anyway. My best friends, Sivert and Vetle, go to this school too, and we play on the same club team. We push each other and compete in a lot of things to make each other better. But sometimes I prefer training on my own to make sure the sessions are effective. It's easy to slack off when you're socialising during training. My motivation is to become the best, and I train really hard because I know I'll get something back from it. I was recently selected for a talent development initiative for the best players in the region. So, you notice that you get something back from the hard work, that it helps to train. But it's not important to be the best now—it's more important to be the best around 20–25.

May 2024

Of course, I'm gutted. I don't understand why I didn't get into the elite upper secondary sports school that picks the best players in the area. Or, that's not entirely true; I know my grades weren't too good. But I reckon the school could have turned a blind eye to that. Sivert and Vetle got in, but they worked hard at school and deserve it, so I try not to be too envious. Anyway, I'll keep working hard and prove them wrong. Right now, I try to, like, listen to my body and make sure I eat enough and drink enough water. Trying to, like … eat right and eat, not necessarily a lot, but eat … my weight could be a bit higher, so I'd benefit from it on the court. Not that I have any issues with eating or anything, but … the calories burn off so quickly that I must eat loads to gain weight. So, I'm trying to eat more like an athlete. I just need to sort out my sleep routine because we've heard since, like, 9th grade—“preferably 9 h each night”—right? It'll help our development and also make it easier for me to concentrate at school.

December 2024

I think the biggest change from lower to upper secondary is the expectation to be self-responsible. In the lower secondary sports school, my teachers kept a close eye on me, but now I'm on my own. “Take responsibility for your own learning”. Easier said than done. Also, I've kind of realised that sport isn't everything, even though it still kind of is. In my new class, almost no one cares if I score ten goals or get selected for some talent development initiatives. At the lower secondary sports school, everyone noticed stuff like that. There, you were cool if you did well in sports, not because of who you hung out with or which parties you got invited to. I miss that. I don't really hang out with my classmates, yet it's still early days, but that's okay because I chill with my teammates, which I prefer anyway.

The thing is, you got to show you can keep it together in more than just one area of life. Honestly, I should’ve paid more attention in school. I guess you could say I'm committed, because I make a lot of the right choices for my sport. I train enough, perform well, take care of myself, and play for a really good club team. Plus, I don't party, which makes me seem more serious compared to others. My new classmates, for example. But I haven't really shown that I'm willing to put all the effort into my education. Truth is, I haven't done a single homework assignment since starting upper secondary school. I keep telling the teachers I'll do it if I have time between trainings, but honestly, there's zero motivation. I'm not really bothered about the grades themselves; it's more about proving to myself that I have enough discipline and can be good at multiple things at once. Especially if handball doesn’t go well sometimes. I want to show myself that I can handle tough times and really work for something important, even when it's difficult.

Theodor positioned himself as a committed athlete through a specific way of knowing performance and development. Across his interviews, a committed athlete was constructed as someone who immersed themself in the pursuit of excellence. As noted in the introduction, a discourse of performance development as an investment constructs an “ideal” figure: the self-regulating, forward-looking athlete who learns to act upon their body in ways that optimise the desired outcomes. This understanding positioned Theodor within a physiological and biomechanical framework of the body, aligning his sense of commitment with disciplinary technologies designed to produce “a training machine”—a biological body capable of sustaining high volumes and intensities of work (24). This construction mirrors patterns documented in numerous studies of youth and elite athletes, where commitment is equated with bodily control, compliance with expert regimes, and a willingness to subordinate other life domains to the long-term pursuit of sporting success [e.g., (56–58)].

During his transition from lower to upper secondary school, Theodor described feeling increased pressure to make responsible choices for his future self. Entering a new environment where athletic achievements were met with indifference, and where his talent status was further destabilised by non-selection to key talent development initiatives, he experienced uncertainty about what it meant to become a successful and autonomous citizen. In response, Theodor adopted visible self-presentation strategies to signal commitment and discipline, such as avoiding parties to differentiate himself from peers and appear more serious about his sporting ambitions. Certainly, he was aware of the public neoliberal ideal that people with a capacity to make self-enhancing investments through education serve as the most qualified actor in modern society (59), but these were clearly public, and not private, values. As such, these efforts can also be interpreted as part of broader negotiations with traditional masculine ideals, including toughness, self-reliance, and a willingness to take risks, which continue to shape the way young men navigate and perform legitimacy in both social and athletic contexts (60).

### 5.2 Elise: at the edges looking in, looking out

In Elise's case, although her physical relocation occurred on a specific date, her transition from a lower secondary sports school to a non-sport upper secondary school unfolded gradually. In the months leading up to the decision, she had already begun reflecting on her desire to meet new people and prioritise her physical wellbeing. While both her family and coaches supported her decision, the transition marked a growing separation between sport and education as distinct, uncoordinated spheres. Elise received no structured support from either her coaches or her new school, and did not request any, leaving her fully responsible for navigating the dual demands. Her efforts to maintain an identity as a committed athlete were increasingly challenged by her club environment, where coaches no longer considered her deserving of a first-team position. Thus, while her educational transition opened space to engage more actively with non-sporting identities, it also disrupted her athletic belonging as she was no longer seen as part of the group most devoted to training.

December 2023

Now that the physiotherapist has figured out what was wrong with my legs, I can participate in almost everything without constantly checking in and worrying. I've had to be careful in training and take it easy quite often, which has been somewhat challenging because you want to participate, and you also want your training to look good when it's assessed at school. But for me, having had so many injuries, I've had to do exercises where I'm almost standing still. It can feel like you're not doing anything. But I love to exercise, and my motivation is really high at the moment. My family is a proper handball family, and my granddad is well proud that I play handball. It's always been like … making him proud, you know, it's a pretty big deal. Since I changed teams, I've made friends for life, and the practices have become more fun and challenging. Handball practices are definitely the most fun, but I also quite like the feeling of getting stronger from the strength training at school. That sense in your body after you've managed to push yourself properly—it's a good feeling.

May 2024

I was pretty chuffed when my coach debunked the myth that you must enrol in a sports school to become a good player, because people often assume that going to a sports school means you're serious about your sport. It gave me more confidence about my choice to apply for an upper secondary media school and focus fully on the club instead. My mum thinks it's a smart move too, given how many injuries I've had recently. Plus, I'm looking forward to changing my environment a bit and being in a class where people have diverse interests. I feel like I need that. I've always believed that if you're going to commit to something, you need to be truly dedicated. For me, it's about being present at every training session, giving it your all, and really showing the coaches that this is something you want. Take the girl on our team who gets up at six in the morning to run before school. She's really good as well, so you can definitely tell she's truly committed. The coaches have said that to be considered for the team, you need to attend all the training sessions. So, I've made sure to let them know whenever I can't make it. I send a message like, “I can’t come because of pollen allergy, we’re sorting a doctor's note,” so they understand. But I do think my coaches know that I want to get somewhere, because one of them is really focused on me and chats a lot with my dad.

November 2024

Lately, I've been feeling a bit demotivated. Everyone's been training lots while I've been ill this autumn, so many of the girls at my team have become really good. I'm still kind of stuck down here while they've developed further. It makes you become a bit, like … see yourself as a bit of a worse player and less valuable compared to the others. Even though the coach said everyone started with a clean slate this season, we [on the second team] can tell he favours the first-team players. I feel like I must show myself more; like, be positive and willing to be there. But, you know, those first-team players, they've got a good connection with the coach, they joke around and chat loads with him and just get plenty of attention from him. So yeah … I'm not entirely sure if I can confidently say that I'm still fully committed. Deep down, yes, I think I am. But right now, I feel uncertain. However, I don't intend to give it up. Besides, life's different now that I've started at an upper secondary media school. At the lower secondary sports school, it was constantly about sport, but now we chat more about parties and boys and stuff. It's really nice going to parties because I can meet more people. So, there's a lot of talk about what we'll do at the weekend and if anyone's got a place we can go to. So yeah, I've made more connections and can ask lots of people to hang out. Our coaches don't care as long as we don't drink or stay up super late before a match, so now I spend most of my free time with friends.

Elise's vignette illustrates a transition shaped by competing discourses and subtle but shifting constructions of what it means to be a committed athlete. Initially, her understanding of commitment was grounded in discourses of fun, intrinsic motivation, and social connection, positioning the committed athlete as someone who trains hard because they genuinely enjoy the activity and value being part of a community (61, 62). These meanings align with a humanistic sports psychology ideal that celebrates autonomy, emotional expression, and positivity (63). However, this construction gradually intersected with performance discourses in which commitment was measured by work ethic, visible effort, and the ability to endure high training loads. Within this logic, motivation became less about joy and more about demonstrating persistence and *performing* commitment, especially for athletes who were not immediately successful and had to “catch up” through visible hard work. Elise's injury experiences were foregrounded to resist dominant culturally accepted standards that equates commitment with volume and physical sacrifice (64), and her orientation towards health partly shaped her decision to leave the sports school pathway. Yet, following her transition, she struggled to meet performance expectations, and her continued emphasis on emotional engagement and relational values over rationalised discipline created tensions in how she saw herself as a committed athlete.

### 5.3 June: why so serious?

In June's case, although she faced a comprehensive transition from junior to senior sport, her educational transition did not result in major overt changes, as both her lower and upper secondary schools were shaped by the same institutional arrangement and organisational culture. She never doubted that she would be accepted into the elite sport school programme, and she expressed confidence that the school would support her academic progression while enabling her to prioritise football. Her coaches and teachers collaborated to create a flexible, tailored schedule that would alleviate the time pressure often experienced by high-performing student-athletes. However, this flexibility had ripple effects, as she spent more time in her club environment and had fewer opportunities to spend time with friends her own age outside the football domain. She described her family as an important resource, noting that her two older siblings had successfully combined sports with higher education, and she often relied on them for support when facing challenges. Her parents never placed pressure on her to perform but instead kept reminding her to have fun with her sport.

December 2023

I still feel like a child, even though I'm playing football at a senior level and it's much more serious. Although there are expectations to perform, it doesn't really get to me, because I understand you can't always play your best. I've always just loved playing with the ball; it's been a fun thing to do with my friends. And it's just grown from there. But it wasn't until I got selected for the youth national team for the first time that I realised I could become quite good. It's all about keeping up the training and focusing on what you can improve and keep having fun. Then you'll keep developing bit by bit. The dream now is to be a senior national team player. I've picked up loads from the older players about how they carry themselves outside of football and how serious they are because they're aiming to succeed. But it's not like it's strict or anything. Things like sleep and eating well just sort of happen naturally. You know it helps you feel better the next day. You want to have energy, and when you're also focusing on school, as it's our last year and we're aiming for good grades, you definitely notice that being well-rested makes a difference. It's not just about training, even though that helps with performance too. Mostly, it's about feeling good overall.

I reckon school is more important than sport, even though I do want to become a professional footballer. I think it's crucial to have something outside of football. Injuries can happen, and I want solid grades to fall back on. That's why the lower secondary sports school has been great. They make adjustments for us, like when we travel with the youth national team and all that. We get schoolwork to do while we're away, so we don't fall too far behind. Plus, the coaches and teachers are always there to help when we need it, so I don't feel like there is too much responsibility, really. They also get that it's important to have a laugh and that it can't be all serious all the time.

December 2024

Last year, I had a lot more responsibility as a sort of leader for the younger ones on the team and with the national team and all that. But this year, I've been more in the background, a bit small, and I've stepped back from taking on that leadership role. At the same time, I've picked up a different kind of responsibility, having to perform well on the pitch. Last year, I wasn't such a key player, but now it feels like I'm expected to play every match. So, the expectations are pretty high … everything you do, the team sort of hinges on it. You can't really make dodgy decisions because the whole team, and the club for that matter, are counting on you to deliver. We train with the club during the day, so my schedule is a bit all over the place. I have to check it to know when I’m actually at school. So yeah, I often catch up on loads of schoolwork, especially on Fridays. But when Sunday rolls around, I try to do as little as possible and just chill.

I make much smarter choices now than I used to. I'm a bit more organised, making sure I'm well-rested and eat enough before training and matches and all that. I reckon it's partly the environment I'm in, too. When everyone else is doing it, it's easier to follow suit. But that doesn't affect me in any negative way, because it's still fun. It all comes quite naturally, and maybe you let loose a bit when there's no match or anything important going on. I don't really feel the need to party, as I reckon it's not great for the body. Plus, as a young athlete, if you drink and mess about, it might signal to the coaches that you're not serious, and then they might not bother to prioritise you. It's almost worse for those of us who are, let's say, at the top of our age group, because people are always looking for something to catch you out on. Anyone who wants your spot and might be a bit jealous could use it against you.

I couldn't manage my education without the support from the teachers at the [elite upper secondary sports school]. For instance, before my midterms, I spent three hours prepping alone with my teacher. They're really flexible and helpful. I can also ask my siblings for help whenever I need to. To be honest, football comes first. It's become much more serious and intense right now. But I still find school really enjoyable, and my parents won't let me prioritise it less, and I don't want to, either. Don’t get me wrong, they think it's proper fun following my football career, but they don't pressure me to do anything. They help me with the mental side of things and, like, let's call it the “drama” in the football environment, like, when everyone except one from the younger players on our team got selected for the national team camp.

Unlike Theodor and Elise, June had already been recognised as a talent and granted access to high-performance environments, including the youth national team and an elite club. Within such contexts, sporting talent is typically associated with a strong commitment to elite sport, a progressive learning trajectory, and aspirations for high-level adult performance (32, 65). These discourses of talent are closely intertwined with performance investment logics that emphasise discipline, work ethic, and goal orientation (66). Yet, June constructed commitment somewhat differently. She conveyed that a committed athlete was someone who is curious, resilient in the face of setbacks, and able to sustain joy and playfulness. She actively mobilised dual career discourses, portraying education and well-being as complementary to athletic success. Notably, her emphasis on “keeping it fun” functioned as a self-governing strategy that helped her manage performance pressures and maintain a sense of balance.

However, as she progressed into more professional environments and secured a starting position on her elite team, June became more entangled in, and voluntarily compliant with, the disciplinary regimes of high-performance sport. Here, commitment was rarely spoken of explicitly but rather enacted through the everyday routines and expectations that surround performance development. Drawing on dominant assumptions that quality training must be efficient, focused, and uninterrupted (67), these environments conferred privilege on athletes with access to superior institutional support, while embedding an implicit moral imperative: not to waste the opportunity. As June herself noted, in such settings, even small deviations from normative behaviours could be interpreted as a lack of seriousness and used against her by coaches and peers.

## 6 Discussion: discursive possibilities for practising commitment

Comparing the “stories” of Theodor, Elise, and June offers valuable insight into the power relations that govern transition processes through secondary school. Each athlete's experience illustrates how subjectivities are shaped within particular institutionalised discourses that define what counts as legitimate knowledge, behaviour, and identity in high-performance sport. All three reproduced elements of dominant performance discourses, supporting the normative construction of the “committed athlete” as someone who successfully manages high volumes of quality training, and who embodies disciplinary values such as work ethics, self-regulation, and accountability [e.g., (19, 68, 69)]. Notably, Elise and June also governed themselves through alternative forms of knowledge, such as fun and motivation, to broaden the meaning of their commitment practices. This could point to the gendered nature of discursive formations in sport, where coaching practices often reproduce normative ideals of masculinity and femininity. Female athletes, in particular, are frequently positioned as more emotionally fragile and in need of “softer” coaching, with feminine enjoyment tied to sociality and cooperation rather than physicality, competitiveness, or excellence (10, 61, 70, 71). These intersecting discourses offer some athletes greater flexibility in how commitment is enacted, while constraining others within narrower, more disciplinary frameworks.

Additionally, the habits and dispositions formed during youth are also shaped by parental expectations and access to economic and cultural capital (72). Drawing on Stefansen and Strandbu (73), it seems that neither Elise nor June experienced an intensive parenting characterised by close monitoring of their athletic development. From a Foucauldian lens, the way parents discursively construct sports participation, and their position as caregivers within these constructions, are central to how athletes become implicated in the disciplinary processes of competitive sports (62, 74).

However, there is a key difference in how Elise and June are positioned within the social hierarchies of their club and team settings. By being considered a talent, June is positioned higher in the hierarchy, which gives her greater discursive authority to construct commitment in less instrumentalised ways. She highlighted that a committed athlete should prioritise learning and development—a discursive right, we argue, primarily preserved to those who can match or surpass the sporting progression of their peers. In contrast, Elise feels obligated to obtain a doctor's note to legitimise her absence from training to coaches; a move signalling her precarious rights to exert power within the performance investment discourse of sport.

From a Foucauldian perspective, this highlights the uneven distribution of discursive power within the web of power relations that characterise talent development systems, and aligns with Foucault's (75) observation that discourse governs not only what can be said and done, but also *who* may act (e.g., June), and *with what authority* (e.g., being a legitimate talent). June's position as a promising talent necessitates her to comply with performance norms, yet it simultaneously affords her the capacity to govern herself through dual career discourses centred on learning and personal growth, preserving her legitimacy within her environment. Elise and Theodor, on the other hand, are interpellated into disciplinary self-governing practices, where their legitimacy hinges on continually proving their commitment, not only to coaches and peers but also to themselves. These are different points of subjectification, shaping how commitment can be understood, negotiated, and enacted within talent—and high-performance sport environments.

By choosing to continue her education outside the sport school system, Elise enacted resistance to the performance discourse that equates increased training volume with better sport development. Interestingly, the fact that her decision required her coach's “permission” underlines the authority of sports school programmes within the current talent development landscape in Norway. Systematically institutionalising the investment ideal, these schools have been established as the “missing link” between systemic requirements for growth and development as well as individual orientations towards a career in high-performance sport (9, 50).

Elise's decision to step away from the dual career pathway came at a cost. No longer embedded within the structured legitimacy of the sport school system, she became more precariously positioned in relation to the regime of truth that defines what counts as legitimate commitment in high-performance sport. This shift exposed her to intensified gaze of evaluation in her club environment, prompting heightened identity management: every training session became a performance in which she had to prove her commitment and justify her continued presence. Although she continued reproducing ways of knowing commitment through the psychological concept of motivation, she lacked the institutional capital and performance markers needed to convincingly live up to coaches' expectations. Her efforts to reconstruct a credible athletic identity ultimately fell short of the norms governing recognition, illustrating how subjectivity in talent development is not merely a matter of effort or attitude, but of discursive legitimacy and unequal access to power, support, and recognition (44).

Building on this, and inspired by Carless and Douglas (25), we see becoming and being talented as performative engagement constituted by the disciplinary requirements of high-performance youth sport environments. Elise's aspiration to work in media, alongside her pursuit of social mobility and diverse relationships, suggests that by stepping away from a system designed, from a Foucauldian perspective, to minimise external distractions and optimise workload management (76), she instead accessed a repertoire of relations to alternative discourses that helped her navigate divergent social identities (39). Nevertheless, it can be a draining project to manage overlapping identities while fighting for recognition from coaches in a landscape characterised by competitiveness and uncertainty (29).

Accepting the institutionalised dual career pathway, Theodor became morally accountable to the individualised knowledge of the performing body, engaging in subjectification processes through which he called upon himself to correct his “weaknesses” in the pursuit of continual performance development. This mirrors how Foucault (77) describes the ways modern subjects render themselves governable. Theodor's practices of self-optimisation, including the regulation of sleep, food, and effort, reflected a sense of himself and his discipline to be, at times, contradictory as he didn't manage to convey such characteristics across various life domains. His difficulty in engaging in alternative ways of understanding or practising commitment can be linked to gendered expectations, as performance discourses in male sporting cultures often intersect with hegemonic constructions of masculinity that normalise an exclusive athletic identity (3, 78).

However, these processes must be seen in relation to a broader “identity work” and discursive power relations, a rationality of rule in which Theodor is confronted with risk scenarios to his future life prospects in the face of educational marginality (28, 79). Within this logic, he is offered only a narrow set of solutions that rest on the promise of meritocratic progression but fail to address the structural conditions that constrain his choices. While performance discourses appear to offer agency, they individualise responsibility and displace attention from the wider network of actors and institutions that shape young people's opportunities and outcomes (32, 80). As Ronkainen and Ryba (81) argue, athletic performance discourses provide a powerful sense of direction for Theodor to “make it in life” outside the educational system. Yet, they may fail to account for critical uncertainties, such as unequal access to opportunities, the role of luck, and the often-overlooked stories of those who do not “make it”.

Theodor's individualised solutions stand in stark contrast to June's transition experiences. From a Foucauldian perspective, June's case illustrates how power operates through normalisation, surveillance, and recognition, granting legitimacy to certain identity positions while rendering other less visible or credible (36). Her early success and alignment with performance metrics secured her a privileged position within the regimes of truth that governs talent development, allowing her commitment to be acknowledged not through constant self-justification, but via institutional validation, including a tailor-made schedule, expert endorsements, and sustained access to high-performance environments. In contrast, athletes like Theodor, who fall outside this network of recognition are denied both material resources (e.g., coaching access) and discursive legitimacy (44). From a relational perspective, these asymmetrical power relations reveal the contingent and unstable nature of agency in the talent development process, where the selection and validation of some athletes inherently destabilises others (32).

These reflections raise important questions about how current holistic development initiatives tend to reinforce performance investment as the central organising principle in young student-athletes' lives. While flexibility is presented as a benefit for student-athletes like June, such flexibility is tightly scripted within pre-structured schedules, which simultaneously serve as mechanisms of control that standardises effectiveness and reinforces the optimised use of time (82). Rather than addressing broader perspectives on well-being or recognising the multiple, performative ways athletes enact commitment, these practices remain focused on ensuring that athletes can meet escalating demands in sport and education without disrupting their training or performance (77). Commitment is thereby reduced to a measurable outcome, something to be optimised and sustained, rather than understood as a negotiated, context-dependent practice.

Extending this critique, the body of scientific knowledge circulating within holistic development practices often reinforce, rather than disrupt, narrow constructions of athletic commitment (4, 83). As our study shows, these initiatives more often benefit student-athletes who can maintain linear developmental trajectories and build trustful relationships with their coaches, while neglecting the ethical dilemmas, tensions, and structural inequities faced by less-performing student-athletes whose lives fall outside the dominant system of thought in high-performance youth sport.

### 6.1 Practical implications and limitations

A central challenge is integrating these ideas into discursive practices that actually allow student-athletes to ask some alternative questions about identities and experiences from multiple standpoints simultaneously (5). Importantly, our work shows that student-athletes reproduce the practices that prioritise commitment as an investment of the self, privileging high performance over other meanings in sport (84). If the aim is to incorporate elements that do not simply teach them to keep their identities separate and maintain them in separate speres but to open the space of talent development to create possibilities for challenging the current norms and values that constrain them, then it is necessary to help student-athletes deconstruct the strong pull of sporting (and academic) practices underpinned by a familiar disciplinary logic (85).

This could include experimenting with different ways of knowing about development to challenge the economised and masculine investment assumptions underlying progress (13). For instance, poststructural and nonlinear coaching pedagogies are gaining attention in academic research, showing how practices, such as disrupting normative truths, decentring coach authority, working with contradiction, uncertainty, or resistance, and creating spaces for marginalised voices or subjugated knowledges, might disrupt traditional disciplinary practices that reinforce top-down expectations (86, 87). In professionalised club environments, there is potential to foster collaboration among stakeholders to engage in critical discussions about how they, often unconsciously, engage in maintaining relationships that promote certain types of committed subjects while devaluing others (15). Educators and institutions could implement workshops to critically interrogate normative assumptions about effective coaching and athletic commitment, helping athletes to identify and address, for instance, how their athletic identities may overshadow or suppress their gendered experiences for reasons of authority and fears of compromising credibility (71, 88). Moreover, creating spaces for dialogue between younger and older athletes during transitions could help the younger athletes to reflect on and create ideas about what “counts” as a possible and desirable life path (2, 3). Offering athletes opportunities to engage with tools that critically explore the emotional and micropolitical dimensions of commitment could serve as an example of how they might begin to question the “cruel optimism”^2^ embedded in sanitised rationale of performance investment and development (89).

The ethical challenge lies in the daunting task of reconstructing the prevailing emphasis on performance within a talent development system built upon dichotomies and clearly defined characteristics of potential. Many young athletes have significantly invested in a self-understanding that affords them privileges and access to resources, and they interpret its rejection as implying inadequacy or a lack of accountability. Furthermore, the repeated search for development holds a meaningful aspect of actively cultivating the physical body to experience one's relation to self and the world differently (90). Therefore, as researchers, we must conceive of power and pleasure, discipline and production, as two intrinsically linked entities [e.g., (38)]. The idea is not to demolish, escape, or try to live outside of the network of relations that facilitate talent development but to participate and enact purposeful changes within it (91).

We purport our study's limitations with privileging analysis of processes of social reproduction, deconstructing discursive possibilities for understanding and practicing commitment. However, through interviews conducted at different points during their transitions and by employing poststructural theory to reconceptualise commitment as relational rather than individual, we were able to trace changes in their discursive resources. This approach is a critical step towards challenging the emphasis on individual responsibility, which obscures structural marginalisation in high-performance youth and senior sport environments. Future research should prioritise researcher-coach collaborations to examine both the short-term and long-term impacts of practices designed to challenge the power/knowledge relations that uphold the investment imperative. As Konoval et al. (92) argue, this requires critically engaging with the educational, ethical, and political articulations that shape our understandings of coaching practices, particularly if we aim to better understand how coaches might exercise power with less domination to foster more ethical practices.

## 7 Conclusion

In this paper, we examined how student-athletes discursively construct and negotiate meanings of commitment during key transitions in education and sport. Using poststructural theory and Foucault's power/knowledge framework, we highlighted how student-athletes come to understand and perform commitment within talent development systems shaped by dominant performance discourses. Our findings underscore the relational and contextual nature of commitment, underlining how these systems tend to privilege the student-athletes who align with established developmental trajectories and foster trustful relationships with their coaches.

While educational programmes in sports schools engage in practices to promote self-regulation, self-empowerment, and responsibility as tools to help young athletes navigate demands in high-performance sport, our study highlights how challenging it is to disrupt dominant discourses of performance investment based on individualised solutions. As such, we argue that supporting student-athletes' ability to broaden their perspectives beyond sport extends beyond reinforcing their autonomy; it requires careful consideration of when, how, and in what contexts coaches can help student-athletes resist pressures of the moral imperative to enact control and manage their body-machine relations to increase their performance development.

## Data Availability

The datasets presented in this article are not readily available because the transcribed interviews is only to be viewed by the first author. Requests to access the datasets should be directed to marielo@nih.no.

## Footnotes

^1^More than 110 Norwegian public and private upper secondary schools now offer elite sport programmes or sports-friendly programmes. Elite programmes are characterised by clear, formal arrangements with clubs and sporting federations, and include daily school training sessions. In contrast, the sports-friendly programmes consist of a three-year general and flexible academic education and five hours of sport training a week during school hours [Sæther, S. A., Feddersen, N., Andresen, E., & Bjørndal, C. T. (2022). Balancing sport and academic development: Perceptions of football players and coaches in two types of Norwegian school-based dual career development environments. *International Journal of Sports Science & Coaching*, *17*(6), 1270–1282. https://doi.org/10.1177/17479541221111462].

^2^Lauren Berlant’s term ‘cruel optimism’ refers to the affective structures that attaches an individual to aspirations, hopes, or goals that seem promising but ultimately undermine their well-being or prevent them from flourishing [Berlant, L. (2011). *Cruel Optimism*. Duke Univesity Press].
